# Lactate dehydrogenase-to-albumin ratio as a potential prognostic indicator in glucocorticoid-treated severe pneumonia: a multicenter retrospective study with external validation

**DOI:** 10.3389/fimmu.2026.1835934

**Published:** 2026-05-13

**Authors:** Jiayi Lin, Jianyi Chen, Zhuolin Liu, Qiuhong Wen, Zetao Zheng, Xueyi Liu, Zijian Zhu, Danfeng Yang, Ziyi Zhou, Dakai Xiao, Chunli Liu

**Affiliations:** 1 State Key Laboratory of Respiratory Diseases, Guangdong Key Laboratory of Vascular Diseases, National Clinical Research Center for Respiratory Diseases, Guangzhou Institute of Respiratory Health, The First Affiliated Hospital of Guangzhou Medical University, Guangzhou, Guangdong, China; 2 Department of Medical Imaging, The Second School of Clinical Medicine, Guangzhou Medical University, Guangzhou, Guangdong, China; 3 Department of Anesthesiology, The Second School of Clinical Medicine, Guangzhou Medical University, Guangzhou, Guangdong, China; 4 Department of Biomedical Engineering, The School of Biomedical Engineering, Guangzhou Medical University, Guangzhou, Guangdong, China; 5 Department of Clinical Medicine, The Second School of Clinical Medicine, Guangzhou Medical University, Guangzhou, Guangdong, China; 6 Department of Clinical Laboratory, National Center for Respiratory Medicine, National Clinical Research Center for Respiratory Disease, State Key Laboratory of Respiratory Disease, Guangzhou Institute of Respiratory Health, The First Affiliated Hospital of Guangzhou Medical University, Guangzhou, Guangdong, China

**Keywords:** glucocorticoid, lactate dehydrogenase-to-albumin ratio, mortality, pneumonia, prognosis, prognostic biomarker

## Abstract

**Background:**

Glucocorticoids are widely used in severe pneumonia but can mask clinical symptoms and traditional severity scores. The lactate dehydrogenase-to-albumin ratio (LAR) reflects the balance between tissue injury and metabolic reserve, yet its prognostic value in steroid-treated pneumonia remains undefined.

**Methods:**

This multicenter study utilized a primary cohort (n=500) and an independent external validation cohort (n=354) of pneumonia patients receiving glucocorticoid therapy. The primary endpoints were 30-day and 90-day all-cause mortality. We employed Cox regression, restricted cubic splines (RCS), and ROC analysis to evaluate LAR performance.

**Results:**

High admission LAR (≥10.48) was identified as a potential prognostic indicator of mortality, associated with a more than twofold risk increase at 30 days (Adjusted HR 2.54; 95% CI: 1.56-4.12) and 90 days (Adjusted HR 2.44; 95% CI: 1.56-3.82). RCS analysis confirmed a non-linear risk escalation with a biological threshold of 10.22 (P non-linearity < 0.001). LAR demonstrated improved predictive discrimination (AUC 0.742) compared to PSI (AUC 0.700) and CURB-65 (AUC 0.654). Although the high LAR group received lower median cumulative glucocorticoid doses (3.0g vs. 5.8g; P < 0.001), this finding was associated with more rapid clinical progression and truncated treatment windows in high-risk patients, reflecting a survival-time-dependent exposure rather than a lack of therapeutic intensity.

**Conclusions:**

Admission LAR is a potential, easily accessible biochemical marker that provides prognostic value independent of typical inflammatory signs masked by steroids. In patients exceeding the 10.22 threshold, our findings suggest a potential efficacy bottleneck for conventional steroid dosing, highlighting a high-risk subpopulation that may require optimized early intervention.

## 1 Introduction

Pneumonia continues to represent a critical global public health threat characterized by substantial mortality (1). Among susceptible populations, patients who require long-term glucocorticoid therapy for chronic airway remodeling diseases or autoimmune disorders face a particularly severe prognosis (2, 3). Chronic glucocorticoid exposure profoundly suppresses host immune defenses and increases vulnerability to respiratory pathogens (4–7). The clinical assessment of these patients is often confounded by radiological and microbiological heterogeneity. Previous evidence suggests that conventional radiography frequently misses non-consolidation infiltrates detectable by CT, especially in sputum culture-negative cases (8). Furthermore, traditional severity scoring systems, such as the Pneumonia Severity Index (PSI) and CURB-65, often underestimate true disease severity in this cohort, as glucocorticoids artificially blunt the critical vital signs and inflammatory symptoms these scores rely upon (9, 10). Given the persistently high mortality and these inherent clinical and diagnostic blind spots, there is an urgent need for accessible, highly sensitive biomarkers to ensure accurate risk stratification and individualized treatment.

Composite biochemical indices derived from routine admission blood tests show significant prognostic value (11, 12). The lactate dehydrogenase-to-albumin ratio (LAR) serves as a key comprehensive marker (13). During severe infection, hypoxia and tissue necrosis trigger a massive release of intracellular lactate dehydrogenase (LDH) (14, 15). Systemic inflammatory cascades also inhibit hepatic albumin synthesis, rapidly depleting this negative acute-phase protein (16, 17). Dynamic changes in LAR reflect the pathophysiological imbalance between severe inflammatory burden and profound nutritional depletion (18, 19). Although previous studies highlight the clinical utility of LAR in severe respiratory diseases, its prognostic potential in glucocorticoid-exposed populations remains unexplored (20, 21). In this specific cohort, traditional prognostic models such as the Pneumonia Severity Index (PSI) and CURB-65 may lose their predictive accuracy, as their reliant vital signs and inflammatory symptoms are easily blunted by the immunosuppressive effects of steroids (22–25). Glucocorticoids exacerbate peripheral protein catabolism to induce iatrogenic hypoalbuminemia and impair macrophage phagocytosis, leading to necrotic tissue accumulation and sustained LDH release. Therefore, we hypothesized that LAR is associated with the immunometabolic status of these patients and may improve risk stratification in scenarios where conventional clinical assessments are affected by glucocorticoid therapy.

Focusing on hospitalized pneumonia patients treated with corticosteroids, we analyzed a retrospective multicenter cohort to determine the prognostic significance of a high admission LAR for 30- and 90-day mortality. We also sought to elucidate the potential non-linear relationship between LAR and mortality risk to establish a precise early-warning clinical cutoff.

## 2 Methods

### 2.1 Data source

This study employed a retrospective observational design with dual independent cohorts. Clinical data for the primary cohort were sourced from the Dryad repository (https://datadryad.org/). This analysis utilized raw clinical profiles obtained from a pneumonia cohort treated at six Chinese secondary and tertiary medical institutions during the 2013–2017 period. The primary investigators implemented rigorous quality control measures, incorporating uniform study protocols and case report forms (CRFs) across all centers, standardized data pre-collection training, and cross-validation of entered data. Drawing on this robust multicenter dataset, the original team previously explored the etiological characteristics and prognostic risk factors among individuals with pneumonia who received glucocorticoid treatments (26).

Data for the external validation cohort were derived from the First Affiliated Hospital of Guangzhou Medical University, a large comprehensive teaching hospital and regional medical center. We retrospectively retrieved consecutive initial cases hospitalized for severe pneumonia at this institution between January 2019 and December 2023, obtaining electronic medical records for a total of 4,213 patients. Baseline clinical information, laboratory indices, and discharge follow-up outcomes for all subjects were systematically extracted and standardized through the hospital’s electronic medical record (EMR) system to independently verify the robustness of the LAR threshold in real-world clinical practice.

This study strictly complied with the ethical guidelines of the Declaration of Helsinki. The data collection and analysis protocol for the external validation cohort received formal approval from the relevant Institutional Review Board (IRB).

### 2.2 Study population and inclusion criteria

**Figure 1:**
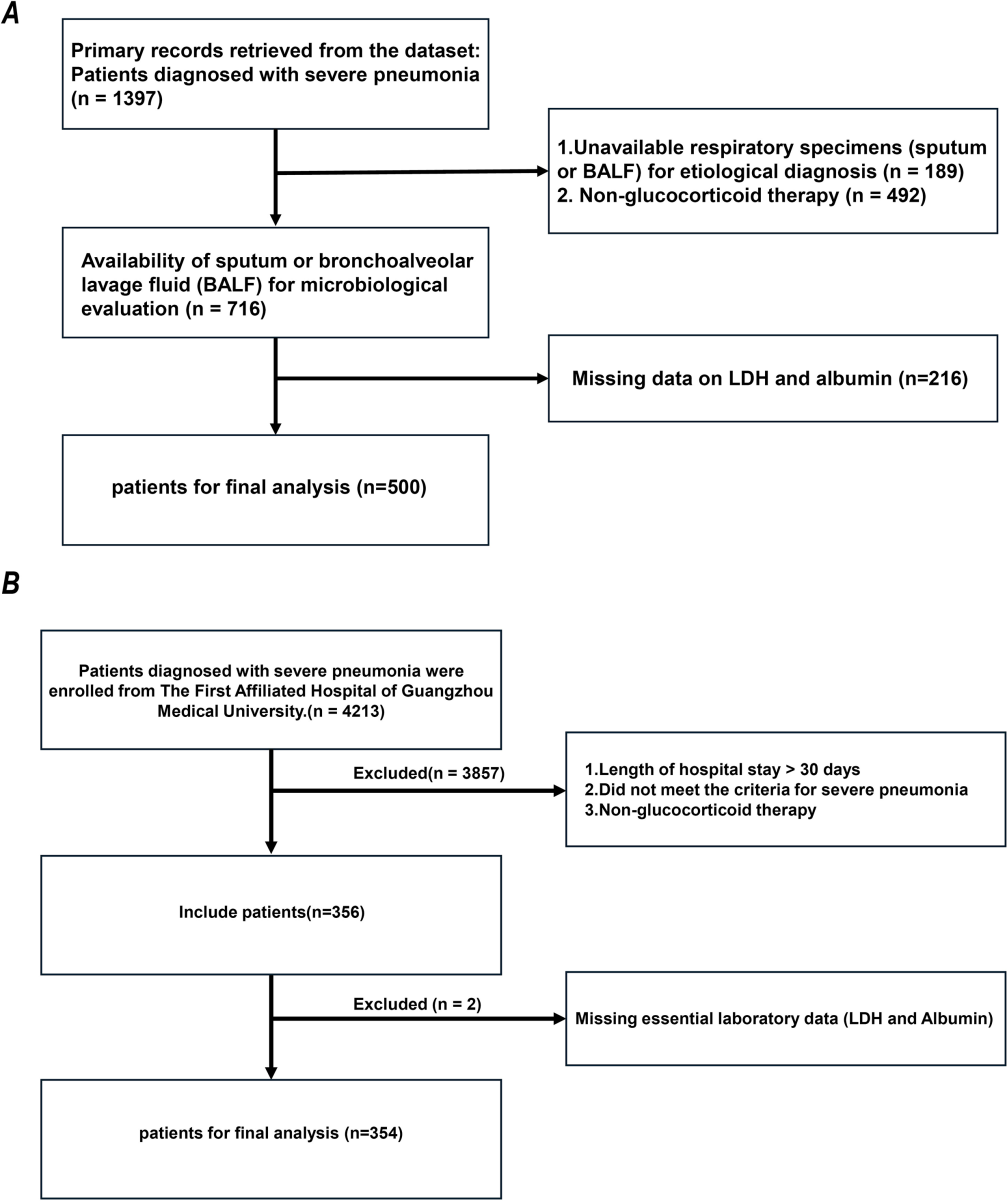
Flowchart of the study cohort. **(A)** Primary cohort. **(B)** External validation cohort.

Pneumonia diagnoses strictly adhered to the official guidelines of the American Thoracic Society (ATS) and the Infectious Diseases Society of America (IDSA). Confirmation required new radiographic infiltrates combined with typical clinical respiratory symptoms or laboratory abnormalities, including fever, new-onset cough, purulent sputum, and abnormal white blood cell counts (27, 28). The primary study included patients aged ≥16 years hospitalized for pneumonia who had received oral or intravenous glucocorticoids prior to admission. Patients with non-infectious pulmonary lesions (e.g., lung cancer, pulmonary embolism, or heart failure) and those without informed consent were previously excluded. For the primary cohort, we further excluded individuals lacking baseline measurements of lactate dehydrogenase (LDH) or serum albumin (ALB) (n = 216). A total of 500 appropriate subjects remained for the downstream statistical analysis of the primary cohort following these comprehensive exclusion steps. **Figure 1A** details the specific selection and exclusion workflow for the primary cohort.

An additional external validation cohort was introduced based on the identical fundamental diagnostic framework, seeking to independently verify the prognostic value and threshold stability of LAR. After confirming that all included individuals had a clear background of glucocorticoid intervention, we similarly excluded cases lacking data for the core baseline indicators (LDH and ALB). A final total of 354 eligible patients with severe pneumonia remained for this validation cohort following the strict screening process. **Figure 1B** details the specific screening workflow for the validation cohort.

### 2.3 Data extraction and LAR

Multidimensional clinical indicators were systematically extracted from electronic medical records. These variables were categorized into six main dimensions: (1) baseline demographics (age and sex); (2) clinical symptoms (fever, cough, and respiratory distress); (3) comorbidity burden (bronchial asthma, chronic obstructive pulmonary disease, coronary artery disease, diabetes mellitus, and chronic renal failure); (4) quantitative severity assessments (Pneumonia Severity Index [PSI]); (5) core hematological and biochemical indices (lactate dehydrogenase, serum albumin, platelet count, aspartate aminotransferase, creatinine, and serum sodium levels); and (6) follow-up outcomes. The cumulative dose of glucocorticoids was standardized to prednisolone equivalents using a standard conversion ratio (5 mg of prednisolone = 5 mg of prednisone = 4 mg of methylprednisolone = 0.75 mg of dexamethasone = 20 mg of hydrocortisone) to evaluate the impact of total steroid exposure on mortality. In our survival analyses, time zero was strictly defined as the day of hospital admission. The timing of exposure measurements for baseline laboratory parameters and clinical severity scores was defined as the initial assessment upon hospital admission (typically within the first 24 hours). Cumulative glucocorticoid doses were calculated based on the total administration during the hospitalization period. The core biomarker, LAR, was defined as the absolute ratio of baseline LDH (U/L) to ALB (g/L). The primary endpoints for the entire cohort were 30-day and 90-day all-cause mortality following admission.

### 2.4 Missing data management

Most baseline covariates in this cohort maintained high data integrity. The cumulative glucocorticoid dose showed a notable missing rate of 13.2% (66/500). **Supplementary Table 1** summarizes the detailed missing rates for all clinical variables. We applied a single imputation technique based on the random forest algorithm to address missing values, aiming to minimize statistical power loss and potential bias from direct deletion. The resulting complete dataset was used for all subsequent statistical analyses and prognostic modeling.

### 2.5 Statistical analysis

Data processing and statistical analyses were performed using R software (version 4.2.3) and Free Statistics software. For the descriptive analysis of baseline characteristics, demographic and clinical parameters of the study population were stratified and summarized according to established LAR levels. Normally distributed continuous variables are presented as mean ± standard deviation (SD). Non-normally distributed continuous variables are reported as median and interquartile range (IQR). Categorical variables are expressed as frequencies and percentages (n, %). We compared continuous baseline variables between groups using the independent t-test or Mann-Whitney U test, depending on data normality. Categorical data were assessed via the chi-square or Fisher’s exact test.

Previous studies indicate that optimal LAR predictive cutoffs vary significantly by primary disease, baseline population characteristics, and illness severity. Given the potential immunological and metabolic heterogeneity of the glucocorticoid-treated population in this cohort, we adopted 10.48 as an *a priori* threshold (29). Subjects were strictly categorized into a low LAR group (< 10.48) and a high LAR group (≥ 10.48) based on this value. We constructed Kaplan-Meier curves to illustrate the effect of abnormal LAR on short- and medium-term mortality. The log-rank test was utilized to evaluate the significance of prognostic differences between groups.

We assessed independent risk factors for mortality by incorporating LAR into Cox proportional hazards regression models as both a continuous and a categorical variable. Model construction followed a progressive stepwise adjustment strategy ensuring robust risk assessment. Model 1 served as an unadjusted crude model. Model 2 controlled for baseline demographics (age, sex) and comorbidity burden (smoking, alcohol consumption, diabetes, etc.). Model 3 included comprehensive adjustments for admission vital signs (temperature, heart rate, respiratory rate, blood pressure), quantitative severity scores (PSI and CURB-65), core interventions (cumulative glucocorticoid dose and vasoactive drugs), and key baseline laboratory parameters (white blood cells, hemoglobin, platelets, AST, ALT, and blood urea nitrogen). We also incorporated restricted cubic spline (RCS) models, flexibly fitting and visualizing potential non-linear dose-response relationships between continuous LAR trajectories and all-cause mortality risk.

Detailed subgroup analyses verified the consistency of our core findings across different clinical subpopulations. Interaction terms in the multivariate Cox models evaluated potential modifying effects between stratification factors and LAR. Given the time-dependent nature of prognostic outcomes, time-dependent ROC curves assessed the predictive discrimination of baseline LAR for 30-day and 90-day mortality. The corresponding time-dependent areas under the curve (AUCs) were compared head-to-head with those of individual LDH and ALB concentrations. A sensitivity analysis was conducted by excluding all individuals with missing covariates and rerunning the multivariate Cox proportional hazards regression models on the complete-case dataset. This approach sought to eliminate potential statistical bias introduced by the single imputation process. Furthermore, to account for potential residual confounding from all other causes other than pneumonia that can be related to LDH increase and albumin decrease, a second sensitivity analysis was performed. In this analysis, we strictly excluded patients diagnosed with specific non-pneumonia-related comorbidities—namely tumors, leukemia, lymphoma, and cirrhosis. We subsequently re-evaluated the associated mortality risk and overall prognostic value of LAR within this purified sub-cohort using the same progressive multivariate Cox proportional hazards regression models to verify the robustness of our primary findings.

An independent external validation cohort was additionally introduced, seeking to further verify the robustness and cross-population generalizability of the independent association between LAR and fatal outcomes. Due to the retrospective nature of the real-world data, specific cumulative exposure doses of glucocorticoids could not be obtained for this cohort. Nevertheless, given that all patients in this cohort possessed a confirmed background of glucocorticoid intervention, and the core objective of the external validation was to examine the independent risk-indicating value of LAR alongside routinely available clinical parameters, a modified multivariate Cox model with streamlined covariates was employed for cross-cohort validation. Model 1 served as an unadjusted crude model. Model 2 controlled for baseline demographic characteristics (age and sex) and core comorbidity burden (smoking, alcohol consumption, coronary heart disease, diabetes mellitus, and connective tissue disease). Model 3 incorporated further in-depth adjustments for core vital signs (heart rate) and key hematological and biochemical indices (white blood cell count, hemoglobin, platelet count, blood urea nitrogen, fibrinogen, and D-dimer). We defined statistical significance as a two-tailed P-value < 0.05 for all analyses.

## 3 Results

### 3.1 Baseline characteristics

The rigorous screening process yielded a final cohort of 500 pneumonia patients receiving glucocorticoid therapy. The 30-day and 90-day all-cause mortality rates in this cohort were 24.0% (n=120) and 27.4% (n=137), respectively. The established LAR threshold of 10.48 stratified participants into two distinct groups. **Table 1** summarizes their detailed baseline clinical characteristics.

**Table 1:** Characteristics of selected participants.

Variables	Total (n = 500)	LAR<10.48 (n = 259)	LAR≥10.48 (n = 241)	p
Gender, n (%)				0.484
Male	255 (51.0)	136 (52.5)	119 (49.4)	
Female	245 (49.0)	123 (47.5)	122 (50.6)	
Age group, n (%)				0.299
<60	239 (47.8)	118 (45.6)	121 (50.2)	
≥60	261 (52.2)	141 (54.4)	120 (49.8)	
Smoke, n (%)				0.843
Never	377 (75.4)	194 (74.9)	183 (75.9)	
Former	104 (20.8)	56 (21.6)	48 (19.9)	
Current	19 (3.8)	9 (3.5)	10 (4.1)	
Alcoholism, n (%)				0.807
No	458 (91.6)	238 (91.9)	220 (91.3)	
Yes	42 (8.4)	21 (8.1)	21 (8.7)	
Diseases, n(%)				
Respiratory Failure				< 0.001
No	243 (48.6)	179 (69.1)	64 (26.6)	
Yes	257 (51.4)	80 (30.9)	177 (73.4)	
COPD				0.022
No	425 (85.0)	211 (81.5)	214 (88.8)	
Yes	75 (15.0)	48 (18.5)	27 (11.2)	
ILD				0.804
No	271 (54.2)	139 (53.7)	132 (54.8)	
Yes	229 (45.8)	120 (46.3)	109 (45.2)	
CHD				0.99
No	442 (88.4)	229 (88.4)	213 (88.4)	
Yes	58 (11.6)	30 (11.6)	28 (11.6)	
DM				0.386
No	373 (74.6)	189 (73)	184 (76.3)	
Yes	127 (25.4)	70 (27)	57 (23.7)	
Hypertension				0.898
No	323 (64.6)	168 (64.9)	155 (64.3)	
Yes	177 (35.4)	91 (35.1)	86 (35.7)	
CRF				0.272
No	465 (93.0)	244 (94.2)	221 (91.7)	
Yes	35 (7.0)	15 (5.8)	20 (8.3)	
CTD				0.092
No	211 (42.2)	100 (38.6)	111 (46.1)	
Yes	289 (57.8)	159 (61.4)	130 (53.9)	
Nephrotic syndrome				< 0.001
No	448 (89.6)	247 (95.4)	201 (83.4)	
Yes	52 (10.4)	12 (4.6)	40 (16.6)	
Tumor				0.828
No	466 (93.2)	242 (93.4)	224 (92.9)	
Yes	34 (6.8)	17 (6.6)	17 (7.1)	
Pneumonia and treatment				
Temperature (°C)	37.4 ± 1.0	37.3 ± 1.0	37.5 ± 1.1	0.02
Heart rate (bpm)	87.8 ± 26.4	85.6 ± 24.0	90.1 ± 28.6	0.055
Respiratory rate (bpm)	22.9 ± 5.4	21.3 ± 3.8	24.5 ± 6.3	< 0.001
Systolic pressure (mmHg)	123.0 ± 19.8	123.6 ± 17.8	122.4 ± 21.7	0.47
Diastolic pressure (mmHg)	74.6 ± 12.6	75.0 ± 11.9	74.1 ± 13.4	0.447
Accumulated dose of glucocorticoids, prednisolone (g)	4.2 (2.2, 8.8)	5.8 (2.8, 13.0)	3.0 (1.8, 6.0)	< 0.001
Vasoactive drugs, n (%)				< 0.001
No	415 (83.0)	238 (91.9)	177 (73.4)	
Yes	85 (17.0)	21 (8.1)	64 (26.6)	
Blood test				
WBC	8.9 ± 5.5	8.1 ± 5.4	9.8 ± 5.6	< 0.001
HGB	113.0 ± 23.1	115.8 ± 24.3	110.0 ± 21.3	0.005
PSI	80.3 ± 31.3	74.3 ± 28.2	86.7 ± 33.1	< 0.001
PLT	189.8 ± 89.5	194.1 ± 83.0	185.1 ± 95.9	0.26
BUN	6.1 (4.6, 9.1)	5.4 (4.1, 7.8)	6.8 (5.2, 10.7)	< 0.001
30-day mortality, n (%)	120 (24.0)	27 (10.4)	93 (38.6)	< 0.001
90-day mortality, n (%)	137 (27.4)	32 (12.4)	105 (43.6)	< 0.001

Data are presented as mean ± SD, median (interquartile range), or n (%).

LAR, lactate-to-albumin ratio; CHD, coronary heart disease; DM, diabetes mellitus; HP, hypertension; CRF, chronic renal failure; CTD, connective tissue disease; COPD, chronic obstructive pulmonary disease; ILD, interstitial lung disease; WBC, white blood cell count; HGB, hemoglobin; PLT, platelet count; BUN, blood urea nitrogen; PSI, pneumonia severity index.

p < 0.05 was considered statistically significant.

Upon admission, patients in the high LAR group exhibited greater clinical severity, characterized by higher body temperatures, elevated respiratory rates, and increased PSI scores. Laboratory findings in this group included significantly elevated levels of WBC and BUN, accompanied by markedly reduced HGB. The prevalence of respiratory failure and COPD was notably higher in this group. These patients also required more frequent administration of vasoactive drugs. Despite receiving a lower median cumulative dose of glucocorticoids, their 30-day and 90-day mortality rates were substantially higher. No significant differences emerged regarding sex, age group, smoking status, or other comorbidities (including CHD, DM, and Hypertension).

### 3.2 In-hospital mortality across different LAR groups

**Figure 2:**
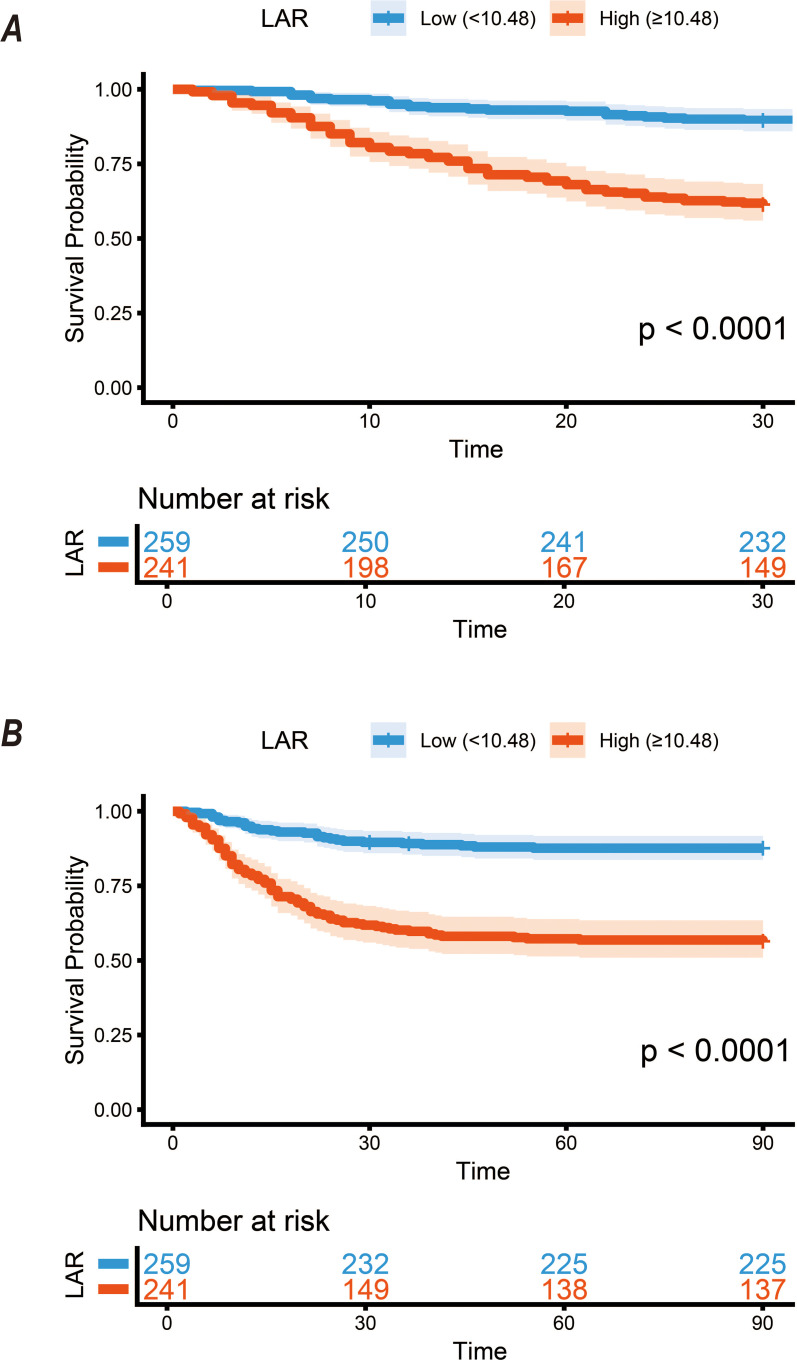
Kaplan-Meier survival curves for all-cause mortality according to the LAR dichotomous numerical classification. **(A)** 30-day and **(B)** 90-day mortality: patients with LAR ≥ 10.48 had significantly lower survival compared with those with LAR<10.48 (P< 0.001).

All-cause mortality during the follow-up period demonstrated highly significant intergroup heterogeneity across different LAR strata. The crude 30-day mortality rate was 10.4% in the low LAR group (< 10.48), contrasting sharply with 38.6% in the high LAR group (≥ 10.48). By day 90, the prognostic disadvantage of the high LAR group became even more pronounced. Their mortality rate (43.6%) reached nearly four times that of the low LAR group (12.4%). Kaplan-Meier survival analysis visually confirmed this trend. The cumulative survival probability exhibited a precipitous decline as baseline LAR levels increased (Log-rank test, P < 0.001; **Figure 2**).

### 3.3 Relationship between LAR and 30-day in-hospital mortality

We present the preliminary univariate findings regarding 30-day mortality for the pneumonia cohort in **Supplementary Table 2**. Subsequent multivariate Cox proportional hazards regression analysis (**Table 2**) confirmed a strong, independent positive correlation between elevated baseline LAR levels and an increased 30-day mortality risk. Our unadjusted analysis (Model 1) evaluating LAR continuously revealed that every one-standard-deviation increment raised the 30-day mortality hazard by 24% (HR = 1.24, 95% CI = 1.15–1.35, P < 0.001). This linear effect remained robust even after comprehensive adjustment for demographics, clinical severity scores, and laboratory parameters in Model 3 (HR = 1.26, 95% CI = 1.10–1.46, P = 0.001).

**Table 2:** Cox regression analysis of LAR and risk of 30-day all-cause mortality.

Variable	N	n.event_%	Model 1		Model 2		Model 3	
			HR (95%CI)	P	HR (95%CI)	P	HR (95%CI)	P
Continuous^a^	500	120 (24)	1.24 (1.15~1.35)	<0.001	1.26 (1.16~1.38)	<0.001	1.26 (1.1~1.46)	0.001
LAR < 10.48	259	27 (10.4)	1(Ref)		1(Ref)		1(Ref)	
LAR ≥ 10.48	241	93 (38.6)	4.45 (2.9~6.84)	<0.001	4.66 (3.02~7.17)	<0.001	2.54 (1.56~4.12)	<0.001

^a^ X was entered as a continuous variable per 1 SD increase.

Model 1: unadjusted.

Model 2: adjusted for age, gender, smoke, alcoholism, DM, CTD, CRF, CHD.

Model 3: adjusted for age, gender, smoke, alcoholism, DM, CTD, CRF, CHD, blood urea nitrogen, temperature, respiratory rate, systolic pressure, diastolic pressure, heart rate, white blood cells, PLT, HGB, CURB-65, PSI, Vasoactive drugs, Accumulated dose of glucocorticoids.

Converting LAR into a categorical variable based on the 10.48 threshold demonstrated even more significant risk stratification efficacy. The crude model revealed that patients in the high LAR group (≥ 10.48) experienced a 4.45-fold higher 30-day mortality risk compared to the control group (HR = 4.45, 95% CI = 2.90–6.84, P < 0.001). The significant positive association between high LAR status and increased 30-day mortality risk persisted robustly following the stringent covariate adjustments in Model 3 (HR = 2.54, 95% CI = 1.56–4.12, P < 0.001).

### 3.4 Relationship between LAR and 90-day in-hospital mortality

**Supplementary Table 2** also presents the univariate Cox regression results for 90-day long-term mortality. Comprehensive results from the multivariate Cox proportional hazards regression models (**Table 3**) confirmed elevated baseline LAR as an independent predictor of poor 90-day prognosis.

**Table 3:** Cox regression analysis of LAR and risk of 90-day all-cause mortality.

Variable	N	n.event_%	Model 1		Model 2		Model 3	
			HR (95%CI)	P	HR (95%CI)	P	HR (95%CI)	P
Continuous^a^	500	137 (27.4)	1.23 (1.14~1.33)	<0.001	1.26 (1.16~1.37)	<0.001	1.23 (1.08~1.41)	0.002
LAR < 10.48	259	32 (12.4)	1 (Ref)		1 (Ref)		1 (Ref)	
LAR ≥ 10.48	241	105 (43.6)	4.36 (2.94~6.49)	<0.001	4.66 (3.13~6.95)	<0.001	2.44 (1.56~3.82)	<0.001

^a^ X was entered as a continuous variable per 1 SD increase.

Model 1: unadjusted.

Model 2: adjusted for age, gender, smoke, alcoholism, DM, CTD, CRF, CHD.

Model 3: adjusted for age, gender, smoke, alcoholism, DM, CTD, CRF, CHD, blood urea nitrogen, temperature, respiratory rate, systolic pressure, diastolic pressure, heart rate, white blood cells, PLT, HGB, CURB-65, PSI, Vasoactive drugs, Accumulated dose of glucocorticoids.

Incorporating LAR as a continuous variable, the crude model (Model 1) indicated a 23% increase in 90-day mortality risk per one-standard-deviation increase (HR = 1.23, 95% CI = 1.14–1.33, P < 0.001). This linear positive correlation remained significant following the extensive covariate control in Model 3 (HR = 1.23, 95% CI = 1.08–1.41, P = 0.002).

Categorical variable analysis based on the 10.48 threshold visually highlighted the long-term lethal burden of high LAR status. Compared to the low LAR group (< 10.48), the high LAR group exhibited a 4.36-fold higher 90-day mortality risk in the unadjusted model (HR = 4.36, 95% CI = 2.94–6.49, P < 0.001). High LAR status maintained its position as a potent independent risk factor for 90-day mortality even after deep adjustment for all potential clinical and biochemical confounders (HR = 2.44, 95% CI = 1.56–3.82, P < 0.001).

### 3.5 RCS analysis of LAR

**Figure 3:**
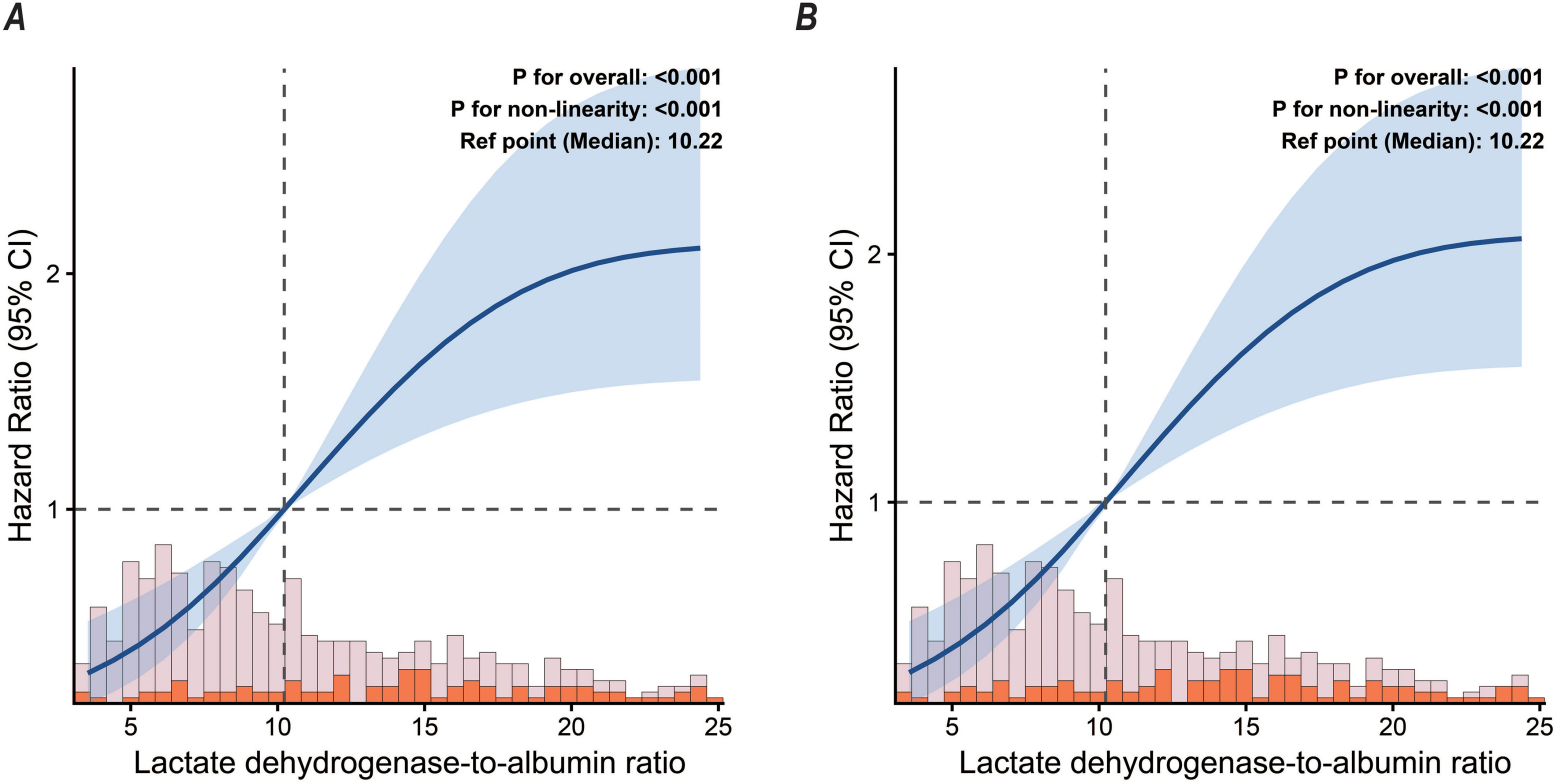
RCS analyses of admission LAR and mortality in pneumonia patients receiving glucocorticoids. **(A)** 30-day in-hospital mortality; **(B)** 90-day in-hospital mortality.

Restricted cubic spline (RCS) analysis followed the comprehensive adjustment for potential confounders. The results revealed a highly significant non-linear positive correlation between LAR levels and both 30-day and 90-day mortality risks (P for overall trend < 0.001, P for non-linearity < 0.001). The model utilized the median LAR of this cohort (10.22) as the reference point (HR = 1.0) for all-cause mortality risk. As illustrated by the smoothing curve in **Figure 3**, the upward trajectory of all-cause mortality risk exhibited a distinct ‘steep-to-gradual’ pattern. Specifically, below this reference point, the hazard ratios (HRs) for both 30-day and 90-day mortality climbed steeply and linearly with increasing LAR. However, once LAR surpassed the 10.22 reference zone, the upward momentum of the risk curve weakened considerably, gradually approaching a flattened plateau. Notably, the striking visual similarity between the 30-day and 90-day hazard ratio curves (Panels A and B) is a direct reflection of the clinical trajectory in this cohort, where the vast majority of fatal events (approximately 88%) occurred during the acute phase (within the first 30 days).

### 3.6 Subgroup analysis

**Figure 4:**
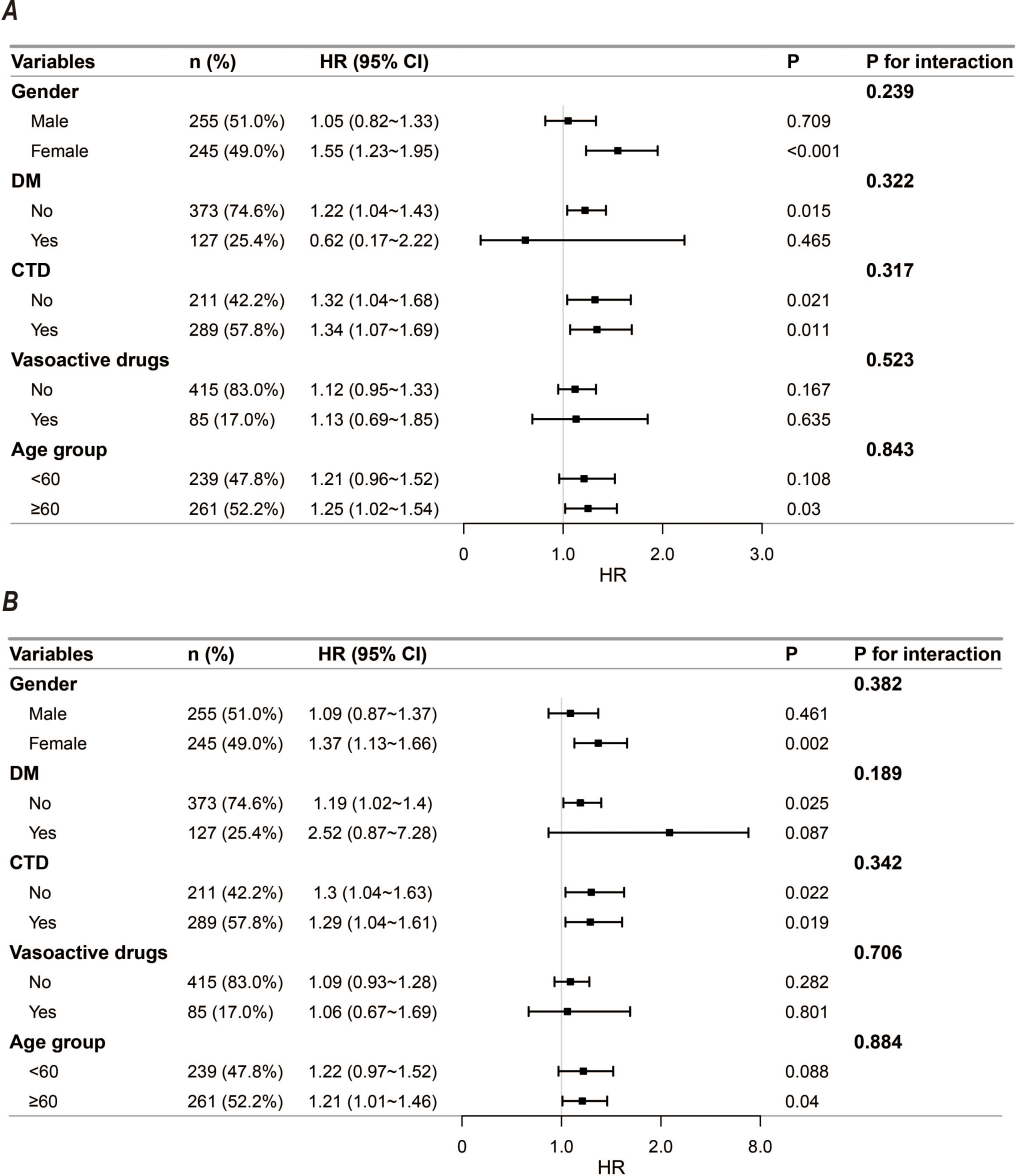
Forest plots of subgroup analyses for the association between baseline LAR and all-cause mortality. **(A)** 30-day in-hospital mortality; **(B)** 90-day in-hospital mortality.

Subgroup and interaction analyses (**Figure 4**) demonstrated no significant modifying effects or statistical interactions between any stratification variables and LAR across all predefined subgroups. These subgroups included sex, underlying comorbidities (such as diabetes mellitus and connective tissue disease), and core therapeutic interventions (all P for interaction > 0.05).

### 3.7 Predictive ability of LAR for in-hospital mortality

**Figure 5:**
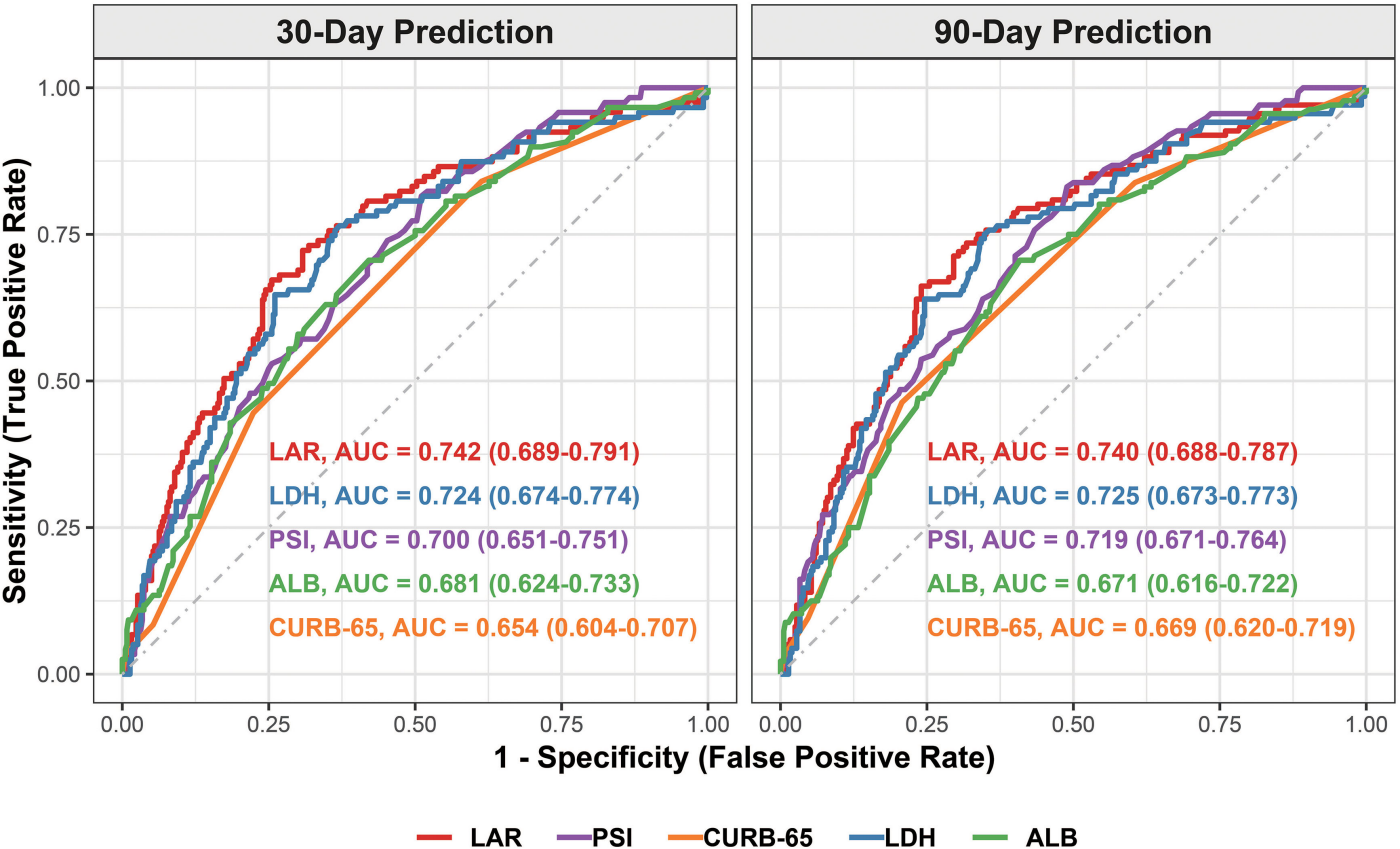
Predictive performance of LAR, individual biomarkers, and clinical scores for all-cause mortality. Time-dependent receiver operating characteristic (ROC) curves comparing the predictive ability of the lactate dehydrogenase-to-albumin ratio (LAR), individual biochemical markers (LDH and ALB), and classic clinical scoring systems (PSI and CURB-65) for 30-day and 90-day all-cause mortality. LAR, lactate dehydrogenase-to-albumin ratio; LDH, lactate dehydrogenase; ALB, albumin; PSI, Pneumonia Severity Index; ROC, receiver operating characteristic.

Receiver operating characteristic (ROC) curve analysis was employed to evaluate the predictive performance of LAR and its individual components (LDH and ALB) for 30-day and 90-day all-cause mortality (**Figure 5**). LAR demonstrated the highest discriminatory capacity for 30-day mortality, yielding an area under the curve (AUC) of 0.742 (95% CI: 0.689 -0.791). This performance numerically outperformed its isolated components, LDH (AUC = 0.724, 95% CI: 0.674 -0.774) and ALB (AUC = 0.681, 95% CI: 0.624 -0.733).

Similarly, LAR maintained superior predictive value for 90-day mortality with an AUC of 0.740 (95% CI: 0.688 -0.787), compared to LDH (AUC = 0.725, 95% CI: 0.673 -0.773) and ALB (AUC = 0.671, 95% CI: 0.616 -0.722). Notably, LAR also displayed superior discriminative capacity over established clinical prognostic scores, including the Pneumonia Severity Index (30-day AUC = 0.700, 95% CI: 0.651 -0.751; 90-day AUC = 0.719, 95% CI: 0.671 -0.764) and the CURB-65 score (30-day AUC = 0.654, 95% CI: 0.604 -0.707; 90-day AUC = 0.669, 95% CI: 0.620 -0.719). These findings underscore the robust efficiency of LAR as an easily accessible biochemical marker for early risk stratification in this patient cohort.

### 3.8 Sensitivity analysis

Sensitivity analysis excluding subjects with incomplete covariate data retained 384 pneumonia patients receiving glucocorticoid therapy. The positive correlation between LAR levels and all-cause mortality remained highly robust within this complete-case cohort. In the fully adjusted model (Model 3), every one-standard-deviation increase in LAR significantly elevated the 30-day and 90-day mortality risks by 33% and 30%, respectively. Crude analysis (Model 1) treating LAR as a categorical variable revealed that patients in the high LAR group (≥ 10.48) faced a 280% higher 30-day mortality risk compared to the low LAR group (< 10.48) (HR = 3.80, 95% CI = 2.38–6.13, P < 0.001; **Supplementary Table 3**). Their 90-day mortality risk was similarly 276% higher (HR = 3.76, 95% CI = 2.42–5.84, P < 0.001; **Supplementary Table 4**). The lethal risk associated with high LAR status remained significantly elevated even after comprehensive adjustment for potential confounders. Model 3 demonstrated a 144% increase in 30-day mortality risk (HR = 2.44, 95% CI = 1.41–4.21, P = 0.001; **Supplementary Table 3**) and a 141% increase in 90-day mortality risk (HR = 2.41, 95% CI = 1.45–3.99, P < 0.001; **Supplementary Table 4**). Furthermore, to robustly confirm that the predictive value of LAR is driven by the pathophysiological cascades of severe pneumonia rather than all other causes other than pneumonia that can be related to LDH increase and albumin decrease, a second sensitivity analysis was conducted by excluding patients with tumors, leukemia, lymphoma, or cirrhosis. Within this strictly purified sub-cohort (n = 456), the independent prognostic value of LAR remained highly consistent. Following comprehensive covariate adjustment (Model 3), high LAR status (≥10.48) continued to act as a potent independent risk factor for mortality, associated with a 181% increase in 30-day mortality risk (HR = 2.81, 95% CI: 1.65–4.77, P < 0.001; **Supplementary Table 5**) and a 163% increase in 90-day mortality risk (HR = 2.63, 95% CI: 1.61–4.27, P < 0.001; **Supplementary Table 6**).

### 3.9 Robustness of the independent association in the external validation cohort

We evaluated the risk stratification value of LAR for 30-day all-cause mortality in an independent external validation cohort (n = 354), seeking to rigorously examine its independent association with in-hospital mortality among severe pneumonia patients receiving glucocorticoid therapy. Notably, a restricted cubic spline (RCS) model was constructed, anchoring 10.48 as the risk reference point (HR = 1.0) (**Supplementary Figure 1**). The results reconfirmed a highly consistent non-linear risk escalation trajectory between LAR levels and 30-day mortality risk within the validation cohort. This dose-response relationship closely mirrored that observed in the primary cohort.

A modified multivariate Cox proportional hazards regression model was subsequently employed to rigorously eliminate the interference of potential clinical confounders (**Table 4**). Incorporating LAR as a continuous variable, the most stringent modified model (Model 3) revealed that every one-standard-deviation (SD) increment independently raised the 30-day mortality risk by 28% (HR = 1.28, 95% CI = 1.13–1.45, P < 0.001). More importantly, categorizing patients based on the *a priori* 10.48 threshold demonstrated the exceptionally robust risk-indicating value of a high LAR status. The unadjusted crude model (Model 1) revealed that patients in the high LAR group (≥ 10.48) experienced a 3.33-fold higher 30-day mortality risk compared to the low LAR group (HR = 3.33, 95% CI = 1.71–6.51, P < 0.001). Even following the most stringent adjustments for baseline demographics, core vital signs, and key laboratory indices in Model 3, crossing the 10.48 risk boundary independently signified a more than twofold increase in the 30-day mortality risk (HR = 2.35, 95% CI = 1.11–5.00, P = 0.026). These independent real-world validation results strongly corroborate the highly robust biological association between elevated LAR and adverse prognosis. They reaffirm the exceptional cross-population generalizability of the 10.48 core risk threshold.

**Table 4:** Cox regression analysis of LAR and risk of 30-day all-cause mortality in the external validation cohort.

Variable	N	n.event_%	Model 1		Model 2		Model 3	
			HR (95%CI)	P	HR (95%CI)	P	HR (95%CI)	P
continuous^a^	354	51 (14.4)	1.25 (1.12~1.39)	<0.001	1.21 (1.05~1.4)	0.007	1.28 (1.13~1.45)	<0.001
LAR < 10.48	159	11 (6.9)	1(Ref)		1(Ref)		1(Ref)	
LAR ≥ 10.48	195	40 (20.5)	3.33 (1.71~6.51)	<0.001	3.25 (1.62~6.52)	<0.001	2.35 (1.11~5)	0.026

^a^ X was entered as a continuous variable per 1 SD increase.

Model 1: unadjusted.

Model 2: adjusted for age, gender, smoke, alcoholism, DM, CTD, CHD.

Model 3: adjusted for age, gender, smoke, alcoholism, DM, CTD, CHD, blood urea nitrogen, heart rate, white blood cells, PLT, HGB, fibrinogen, and D-dimer.

## 4 Discussion

Utilizing the multicenter Dryad repository, this retrospective cohort investigation evaluated the prognostic utility of the lactate dehydrogenase-to-albumin ratio (LAR) among hospitalized pneumonia patients undergoing corticosteroid therapy. The findings suggest a potential, independent positive correlation between abnormally elevated baseline LAR and a sharp increase in both 30-day and 90-day all-cause mortality risks. Patients in the high LAR cohort experienced mortality rates of 38.6% and 43.6% at days 30 and 90, respectively. These proportions overwhelmingly exceeded the 10.4% and 12.4% baseline mortality rates observed in the low LAR population. Comprehensive model adjustment for potential clinical and biochemical confounding variables confirmed high LAR status as a potent independent risk factor, increasing the 30-day and 90-day mortality risks by more than twofold (HR = 2.54 and 2.44, respectively). Receiver operating characteristic modeling confirmed that the combined LAR metric delivers enhanced discriminatory power for forecasting early and intermediate lethal events, significantly outperforming individual LDH or ALB evaluations. Restricted cubic spline analysis demonstrated a pronounced curvilinear and threshold-driven relationship linking LAR to mortality risk. The median risk reference point (10.22) derived from our real-world data demonstrated striking consistency with the optimal cutoff value (10.48) established in previous literature (29). In an independent external validation cohort, we successfully replicated this highly consistent non-linear risk escalation trajectory and independent lethal effect using threshold-anchored multivariate Cox regression and RCS models. This convergence of thresholds and replication of efficacy across diverse real-world clinical cohorts not only firmly solidifies the rationale of our grouping strategy but also strongly demonstrates the exceptional cross-population generalizability and absolute statistical robustness of LAR as an early warning indicator for severe illness.

The ‘dosage paradox’ observed in **Table 1**—where patients with higher LAR and higher mortality received lower median accumulated doses (3.0g vs. 5.8g)—merits careful interpretation. This phenomenon likely represents a statistical guarantee-time bias (also known as immortal time bias) rather than a lack of therapeutic intensity. As illustrated by the Kaplan-Meier curves in **Figure 2**, high LAR patients often faced an accelerated clinical deterioration, with a precipitous decline in survival within the first 14 days. This early mortality naturally truncated the observation and treatment windows, preventing these patients from completing a full glucocorticoid tapering cycle. Consequently, in this retrospective context, the cumulative dose serves as a proxy for survival duration. This finding reinforces the concept that high LAR identifies a ‘futility window’ for standard steroid therapy, where patients reach an efficacy bottleneck or metabolic collapse before the intended treatment can be fully realized.

The current study pioneers the comprehensive evaluation of LAR as a prognostic indicator specifically in pneumonia patients with long-term glucocorticoid exposure. Previous literature highlights the clinical value of LAR across various conditions, including severe community-acquired pneumonia, neoplastic diseases, and COVID-19 (13, 21, 29). This composite marker typically reflects a systemic pathophysiological imbalance between severe inflammatory responses and profound nutritional depletion (30, 31).

Alongside LAR, several conventional composite biochemical indices and classic biomarkers are frequently utilized for risk stratification in severe pulmonary infections. For instance, the blood urea nitrogen-to-albumin ratio (BAR) has shown utility in predicting mortality and the necessity for intensive care (32–34). BAR integrates the metabolic consequences of systemic inflammation with renal hypoperfusion and hydration status (33). Similarly, the lactate dehydrogenase-to-lymphocyte ratio (LLR) serves as an integrated indicator of pulmonary parenchymal damage and systemic inflammatory insult, coupling the metabolic burden of necrotic cell death with the immunological exhaustion observed in severe pneumonia (35–37). Furthermore, classic acute-phase reactants and immunological markers, including C-reactive protein (CRP), procalcitonin (PCT), and the neutrophil-to-lymphocyte ratio (NLR), serve as standard clinical references for evaluating systemic inflammatory drive and innate immune imbalance (38–41). These conventional metrics serve as accessible clinical references for assessing systemic responses and metabolic disturbances during severe infections.

However, the prognostic performance of these traditional metrics may be influenced in patients receiving glucocorticoid therapy. Glucocorticoids can accelerate hepatic urea synthesis and increase systemic nitrogen load, which can lead to elevated blood urea nitrogen (BUN) levels, thereby potentially affecting the predictive accuracy of BAR (42, 43). Furthermore, corticosteroids exert potent lympholytic effects by promoting the apoptosis and margination of circulating lymphocytes, which can lead to iatrogenic lymphocytopenia and variations in the LLR (44, 45). Coupled with glucocorticoid-induced non-infectious neutrophilia, these lympholytic effects also severely distort the baseline NLR (44). Beyond these cellular shifts, glucocorticoids suppress the transcription of pro-inflammatory cytokines, artificially blunting the synthesis of CRP independent of the true infectious burden (46, 47). Although procalcitonin (PCT) exhibits relative resistance to this steroid-induced suppression, its prognostic scope primarily reflects bacterial burden rather than the systemic metabolic exhaustion of the host (48–50).

Consequently, the present research expands the clinical applicability of LAR to highly susceptible, immunocompromised populations. This expansion underscores the robust prognostic utility of this indicator, even when the pharmacological effects of glucocorticoids attenuate or distort typical inflammatory and metabolic profiles.

The potential biological mechanism of LAR as a prognostic indicator stems from its capacity to concurrently reflect inflammation-driven tissue necrosis and systemic metabolic depletion during severe infection. Glucocorticoid intervention significantly influences both pathological processes. Within the tissue microenvironment, ubiquitous cytoplasmic LDH rapidly enters the bloodstream following the compromise of cell membrane integrity (51–53). Alveolar epithelial cell damage, microvascular thrombosis, and local tissue hypoxia in severe pneumonia force cellular metabolism to reprogram toward anaerobic glycolysis, triggering massive LDH synthesis and release (54–56). Hyperactivated immune cells undergoing necrosis or pyroptosis at inflammatory sites release substantial intracellular enzymes into the circulation (52, 57). Elevated serum LDH serves as a direct biochemical manifestation of progressive pulmonary parenchyma destruction and microcirculatory ischemia (58). Continuous local tissue disintegration closely intertwines with the overall metabolic state (59). Hepatic synthesis of ALB, a negative acute-phase protein, declines significantly during severe infection (60, 61). Systemic inflammation-induced vascular endothelial barrier dysfunction and capillary leakage contribute to this downward trend. Massive proinflammatory cytokines also strongly inhibit hepatic albumin gene transcription (62–64). Albumin maintains plasma colloid osmotic pressure and acts as a natural antioxidant and toxin carrier in the blood (65, 66). Its rapid depletion signals the entry into a profound negative nitrogen balance and compromised antioxidant defense systems (67, 68). This state exacerbates oxidative stress-mediated damage to the microcirculatory endothelium and target organs (69, 70).

Glucocorticoid therapy exacerbates this pathophysiological imbalance. Immunosuppressive effects promote peripheral protein catabolism, accelerating iatrogenic albumin depletion (71, 72). Impaired macrophage function disrupts immune surveillance, fueling persistent local cellular destruction and sustained LDH release (73, 74). Continuous tissue disintegration parallels accelerated defensive protein depletion, further exacerbating the patient’s critical condition. Abnormally elevated LAR serves as a reliable indicator of this state. The ratio clearly indicates infection-mediated tissue destruction overwhelming the host’s nutritional and physiological reserves (75, 76).

This study confirms LAR as a highly innovative, accessible, and cost-effective prognostic evaluation tool for pneumonia patients receiving glucocorticoid intervention. This simplified biochemical ratio exhibited predictive superiority over the standard CURB-65 score and the complex 20-variable PSI within our study cohort. This confirms a crucial clinical benefit for rapid decision-making in emergency and critical care environments. For the highly heterogeneous group of severe pneumonia patients receiving glucocorticoids, LAR serves as a highly cost-effective and immediate triage alternative, eliminating the need for time-consuming clinical evaluations without compromising prognostic accuracy.

While classic scores like PSI and CURB-65 typically achieve predictive AUCs above 0.75 to 0.80 in broad pneumonia populations, their efficacy significantly diminished in our specific glucocorticoid-intervention cohort (9, 10). Beyond potential selection biases inherent to retrospective studies, we hypothesize that this performance gap primarily reflects the distinct pathophysiology of our target subjects. The potent immunosuppressive and anti-inflammatory properties of glucocorticoids can significantly obscure the initial clinical manifestations of severe infection, artificially blunting critical warning signs such as fever and tachycardia (24, 25, 77). Relying heavily on these suppressed vital signs makes PSI and CURB-65 highly susceptible to severe clinical underestimations here (22, 23). Conversely, by directly quantifying tissue microcirculatory breakdown and systemic negative nitrogen balance, LAR remains independent of this hormone-blunted presentation. This mechanistic advantage enables LAR to overcome the masking effects of steroids, demonstrating robust discriminatory power for identifying high-risk patients that conventional metrics might otherwise miss. Furthermore, it should be emphasized that in clinical practice, LAR is conceptualized as a rapid, standalone surrogate indicator for acute risk stratification, rather than an additive parameter designed to augment existing multivariable scoring systems.

Subgroup evaluation comprehensively verified the cross-population robustness of LAR’s prognostic efficacy. Sex, chronic comorbidities such as hypertension and diabetes mellitus (DM), and core intervention strategies exhibited no significant modifying effects or statistical interactions on the mortality risk driven by elevated baseline LAR. This highly consistent risk prediction trajectory greatly expands the clinical applicability boundaries of LAR within complex immunosuppressed cohorts. Patients on long-term glucocorticoid therapy often present with profound metabolic disturbances and masked conventional infection signs. Abnormal admission LAR levels effectively penetrate the interference of these heterogeneous clinical manifestations. High LAR consistently and precisely anchors potential lethal outcomes across diverse scenarios, from evaluating susceptible individuals with superimposed metabolic diseases to assessing critically ill patients at various disease progression stages. This finding firmly establishes this core ratio as a universal early warning tool in real-world clinical practice.

Several important limitations must be acknowledged. The retrospective observational design inherently precludes the establishment of definitive causal inferences. Despite rigorous multivariate adjustments for key confounders, including PSI scores, the possibility of residual confounding cannot be entirely eliminated. Specifically, database limitations prevented the acquisition of mainstream inflammatory biomarkers, such as interleukin-6, precluding parallel comparisons. Simultaneously, the datasets lacked specific pharmacological details regarding glucocorticoid interventions, encompassing treatment duration, tapering schedules, and administration routes, as well as cause-specific mortality data capable of differentiating refractory infection complications from underlying disease exacerbation. Crucially, the highly heterogeneous cumulative doses of glucocorticoids pose a risk of confounding by indication, as patients with varying disease trajectories likely received different dosing regimens. Incomplete retrospective medical documentation at the external validation center also hindered the extraction of the complex parameters necessary for PSI and CURB-65 calculations, precluding a head-to-head performance comparison within this cohort. Paradoxically, this objective data acquisition barrier reinforces our core thesis: unlike traditional scoring tools highly dependent on numerous cumbersome physiological metrics, the LAR requires only routine biochemical assays, thereby offering unparalleled accessibility and practical utility in demanding real-world emergency settings. Future prospective multicenter cohort studies urgently need to incorporate continuous dynamic LAR measurements and comprehensive clinical treatment data. These investigations will further validate the 10.22 non-linear inflection point and optimize the ideal threshold for precision medical guidance.

The dosage paradox identified in **Table 1** uncovers an important clinical consideration: in retrospective studies of severe pneumonia, the accumulated therapeutic dose is often a derivative variable of survival time rather than a reflection of treatment intensity alone. The LAR biomarker helps identify vulnerable patients who face an elevated risk of mortality and who may exhibit a diminished response to conventional steroid interventions. For clinicians, admission LAR monitoring should be regarded as a critical early-warning signal rather than merely a routine laboratory test. When LAR ≥ 10.48, it suggests that the patient’s clinical risk may exceed what is captured by traditional scoring systems; instead of focusing on mid-course dose accumulation, optimizing early interventions shortly after admission. Future research should explore whether alternative or adjunctive early immunomodulatory strategies can effectively reduce LAR and improve survival trajectories for patients crossing the 10.48 threshold. The application of LAR signifies a potential shift from vital sign-driven to biochemical essence-driven management, providing preliminary evidence for precision stratification in severe pneumonia.

## 5 Conclusion

In conclusion, this multicenter retrospective study suggests that admission LAR may serve as a potential and easily accessible prognostic indicator for pneumonia patients receiving glucocorticoid therapy. Elevated baseline LAR was found to be independently associated with an increased risk of mortality, potentially reflecting both inflammation-driven cellular injury and systemic metabolic exhaustion. While LAR may offer a useful supplementary tool for risk stratification in these high-risk patients, clinicians should interpret these findings within the context of existing clinical scores. As an observational study, these results should be considered hypothesis-generating; therefore, large-scale prospective validation is strictly required before the clinical implementation of LAR-guided intervention strategies can be recommended.

## Data Availability

The datasets presented in this study can be found in online repositories. The names of the repository/repositories and accession number(s) can be found below: the data that support the findings of this study are openly available in Dryad at https://doi.org/10.5061/dryad.mkkwh70x2. The datasets for the external validation cohort will be made available upon request pending application and approval by Chunli Liu, chunli@gird.cn.
