# Supplementary material for: Lactate dehydrogenase-to-albumin ratio as a potential prognostic indicator in glucocorticoid-treated severe pneumonia: a multicenter retrospective study with external validation
Source: Front Immunol. 2026 May 13;17:1835934. doi: 10.3389/fimmu.2026.1835934 (PMC13212226; doi:10.3389/fimmu.2026.1835934)
Supplement: Supplementary file 1 [file DataSheet1.docx]

Supplementary Material

# Supplementary Figures

**Figure S1. RCS analysis of admission LAR and 30-day mortality in glucocorticoid-treated pneumonia validation cohort.**


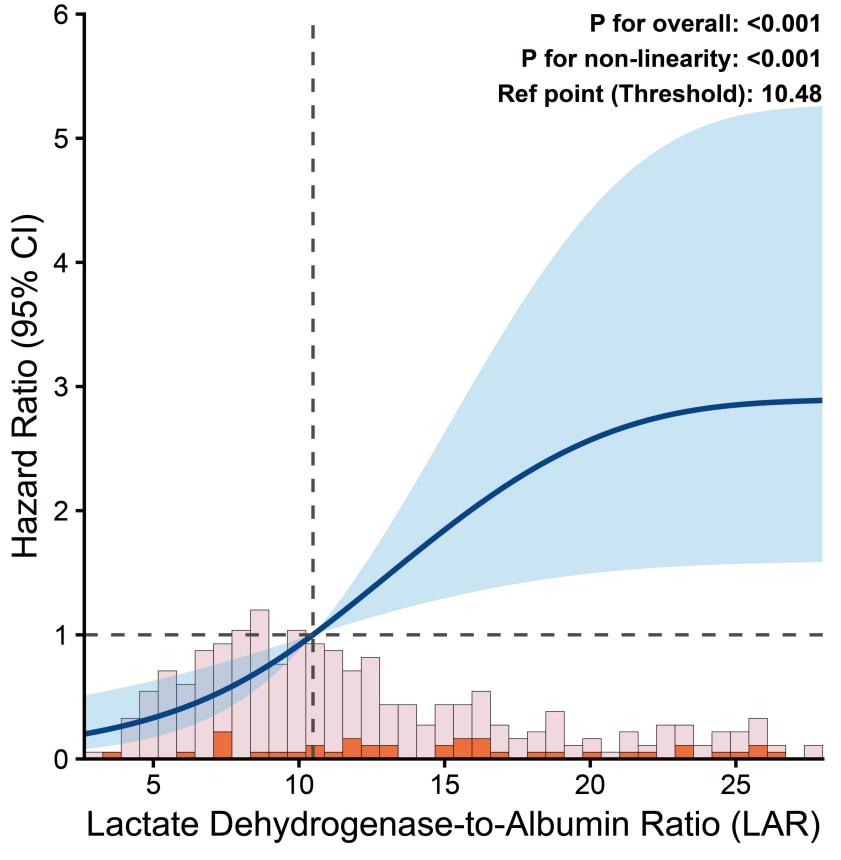


# Supplementary Tables

**Table S1. Details of missing data**

| Variable | Missing Count | Missing Percent |
| --- | --- | --- |
| Accumulated dose of glucocorticoids | 66 | 13.20% |
| Temperature | 33 | 6.60% |
| PLT | 10 | 2% |
| WBC | 3 | 0.60% |
| HGB | 3 | 0.60% |
| BUN | 1 | 0.20% |
| 30-day mortality | 0 | 0% |
| 90-day mortality | 0 | 0% |
| LAR | 0 | 0% |
| Age | 0 | 0% |
| Gender | 0 | 0% |
| CHD | 0 | 0% |
| DM | 0 | 0% |
| CRF | 0 | 0% |
| CTD | 0 | 0% |
| Smoke | 0 | 0% |
| Alcoholism | 0 | 0% |
| Heart rate | 0 | 0% |
| Respiratory rate | 0 | 0% |
| Systolic pressure | 0 | 0% |
| Diastolic pressure | 0 | 0% |
| Curb65 | 0 | 0% |
| PSI | 0 | 0% |
| Vasoactive drugs | 0 | 0% |

Abbreviations: PLT, platelet count; WBC, white blood cell count; HGB, hemoglobin; BUN, blood urea nitrogen; LAR, lactate-to-albumin ratio.

**Table S2. Results of univariate cox regression analysis of 30-day and 90-day mortality**

| Variables | 30d-mortality | | 90d-mortality | |
| --- | --- | --- | --- | --- |
|  | HR (95%CI) | *P-value* | HR (95%CI) | *P-value* |
| Age | 1.13 (1.00, 1.29) | 0.049 | 1.17 (1.04, 1.32) | 0.01 |
| Gender | 0.87 (0.61, 1.25) | 0.446 | 0.85 (0.60, 1.18) | 0.33 |
| CHD | 1.34 (0.80, 2.24) | 0.264 | 1.39 (0.87, 2.24) | 0.171 |
| DM | 0.97 (0.64, 1.46) | 0.866 | 1.08 (0.74, 1.57) | 0.696 |
| CRF | 1.21 (0.63, 2.32) | 0.559 | 1.17 (0.63, 2.17) | 0.612 |
| CTD | 0.95 (0.66, 1.36) | 0.76 | 1.05 (0.75, 1.48) | 0.777 |
| Smoke | 1.32 (0.97, 1.80) | 0.081 | 1.33 (0.99, 1.78) | 0.057 |
| Alcoholism | 1.43 (0.81, 2.55) | 0.221 | 1.34 (0.77, 2.33) | 0.296 |
| Temperature | 1.26 (1.07, 1.48) | 0.005 | 1.27 (1.10, 1.48) | 0.002 |
| Heart rate | 1.00 (1.00, 1.01) | 0.238 | 1.00 (0.99, 1.01) | 0.639 |
| Respiratory rate | 1.05 (1.02, 1.07) | < 0.001 | 1.05 (1.03, 1.08) | < 0.001 |
| Systolic pressure | 1.00 (0.99, 1.01) | 0.651 | 1.00 (0.99, 1.01) | 0.737 |
| Diastolic pressure | 1.00 (0.99, 1.01) | 0.932 | 1.00 (0.98, 1.01) | 0.731 |
| WBC | 1.04 (1.02, 1.06) | < 0.001 | 1.04 (1.02, 1.06) | < 0.001 |
| HGB | 0.99 (0.99, 1.00) | 0.109 | 0.99 (0.99, 1.00) | 0.042 |
| Curb65 | 1.62 (1.36, 1.93) | < 0.001 | 1.68 (1.43, 1.97) | < 0.001 |
| PSI | 1.02 (1.01, 1.02) | < 0.001 | 1.02 (1.01, 1.02) | < 0.001 |
| Accumulated dose of glucocorticoids. | 0.98 (0.96, 1.00) | 0.028 | 0.97 (0.95, 0.99) | 0.011 |
| PLT | 1.00 (0.99, 1.00) | < 0.001 | 1.00 (0.99, 1.00) | < 0.001 |
| Vasoactive drugs | 8.19 (5.69, 11.77) | < 0.001 | 9.59 (6.80, 13.52) | < 0.001 |
| BUN | 1.06 (1.04, 1.08) | < 0.001 | 1.06 (1.04, 1.07) | < 0.001 |

Note: Data are presented as HRs and 95% CIs.

Abbreviations: HR, hazard ratio; CI, confidence interval; CHD, coronary heart disease; DM, diabetes mellitus; CRF, chronic renal failure; CTD, connective triangle disease; WBC, white blood cell count; HGB, hemoglobin; PLT, platelet count; BUN, blood urea nitrogen; PSI, pneumonia severity index.

**Table S3. Cox proportional hazard ratios (HRs) for 30 days all-cause mortality**

| Variable | N | n.event_% | Model 1 | | Model 2 | | Model 3 | |
| --- | --- | --- | --- | --- | --- | --- | --- | --- |
|  |  |  | HR (95%CI) | P | HR (95%CI) | P | HR (95%CI) | P |
| continuous^a^ | 394 | 94 (23.9) | 1.33 (1.19~1.47) | <0.001 | 1.45 (1.25~1.68) | <0.001 | 1.33 (1.10~1.60) | 0.003 |
| LAR < 10.48 | 196 | 22 (11.2) | 1(Ref) |  | 1(Ref) |  | 1(Ref) |  |
| LAR ≥ 10.48 | 199 | 72 (36.4) | 3.80 (2.376~6.13) | <0.001 | 3.88 (2.39~6.31) | <0.001 | 2.44 (1.41~4.21) | 0.001 |

^a^ X was entered as a continuous variable per 1 SD increase.

Model 1: unadjusted.

Model 2: adjusted for age, gender, smoke, alcoholism, DM, CTD, CRF, CHD.

Model 3: adjusted for age, gender, smoke, alcoholism, DM, CTD, CRF, CHD, blood urea nitrogen, temperature, respiratory rate, systolic pressure, diastolic pressure, heart rate, white blood cells, PLT, HGB, CURB-65, PSI, Vasoactive drugs, Accumulated dose of glucocorticoids.

**Table S4. Cox proportional hazard ratios (HRs) for 90 days all-cause mortality**

| Variable | N | n.event_% | Model 1 | | Model 2 | | Model 3 | |
| --- | --- | --- | --- | --- | --- | --- | --- | --- |
|  |  |  | HR (95%CI) | P | HR (95%CI) | P | HR (95%CI) | P |
| continuous^a^ | 395 | 108 (27.4) | 1.31 (1.18~1.45) | <0.001 | 1.43 (1.25~1.64) | <0.001 | 1.3 (1.09~1.55) | 0.003 |
| LAR < 10.48 | 196 | 26 (13.3) | 1(Ref) |  | 1(Ref) |  | 1(Ref) |  |
| LAR ≥ 10.48 | 199 | 82 (41.4) | 3.76 (2.42~5.84) | <0.001 | 3.97 (2.53~6.23) | <0.001 | 2.41 (1.45~3.99) | 0.001 |

^a^ X was entered as a continuous variable per 1 SD increase.

Model 1: unadjusted.

Model 2: adjusted for age, gender, smoke, alcoholism, DM, CTD, CRF, CHD.

Model 3: adjusted for age, gender, smoke, alcoholism, DM, CTD, CRF, CHD, blood urea nitrogen, temperature, respiratory rate, systolic pressure, diastolic pressure, heart rate, white blood cells, PLT, HGB, CURB-65, PSI, Vasoactive drugs, Accumulated dose of glucocorticoids.

**Table S5. Sensitivity analysis: Cox proportional hazard ratios (HRs) for 30-day all-cause mortality (excluding patients with tumors, leukemia, lymphoma, or cirrhosis)**

| Variable | N | n.event_% | Model 1 | | Model 2 | | Model 3 | |
| --- | --- | --- | --- | --- | --- | --- | --- | --- |
|  |  |  | HR (95%CI) | P | HR (95%CI) | P | HR (95%CI) | P |
| continuous^a^ | 456 | 111 (24.3) | 1.25 (1.15~1.35) | <0.001 | 1.26 (1.15~1.38) | <0.001 | 1.27 (1.1~1.46) | 0.001 |
| LAR < 10.48 | 237 | 23 (9.7) | 1(Ref) |  | 1(Ref) |  | 1(Ref) |  |
| LAR ≥ 10.48 | 219 | 88 (40.2) | 5.01 (3.16~7.93) | <0.001 | 5.2 (3.27~8.26) | <0.001 | 2.81 (1.65~4.77) | <0.001 |

^a^ X was entered as a continuous variable per 1 SD increase.

Model 1: unadjusted.

Model 2: adjusted for age, gender, smoke, alcoholism, DM, CTD, CRF, CHD.

Model 3: adjusted for age, gender, smoke, alcoholism, DM, CTD, CRF, CHD, blood urea nitrogen, temperature, respiratory rate, systolic pressure, diastolic pressure, heart rate, white blood cells, PLT, HGB, CURB-65, PSI, Vasoactive drugs, Accumulated dose of glucocorticoids.

**Table S6. Sensitivity analysis: Cox proportional hazard ratios (HRs) for 90-day all-cause mortality (excluding patients with tumors, leukemia, lymphoma, or cirrhosis)**

| Variable | N | n.event_% | Model 1 | | Model 2 | | Model 3 | |
| --- | --- | --- | --- | --- | --- | --- | --- | --- |
|  |  |  | HR (95%CI) | P | HR (95%CI) | P | HR (95%CI) | P |
| continuous^a^ | 456 | 128 (28.1) | 1.23 (1.14~1.34) | <0.001 | 1.26 (1.15~1.37) | <0.001 | 1.24 (1.08~1.41) | 0.002 |
| LAR < 10.48 | 237 | 28 (11.8) | 1(Ref) |  | 1(Ref) |  | 1(Ref) |  |
| LAR ≥ 10.48 | 219 | 100 (45.7) | 4.84 (3.18~7.37) | <0.001 | 5.2 (3.4~7.94) | <0.001 | 2.63 (1.61~4.27) | <0.001 |

^a^ X was entered as a continuous variable per 1 SD increase.

Model 1: unadjusted.

Model 2: adjusted for age, gender, smoke, alcoholism, DM, CTD, CRF, CHD.

Model 3: adjusted for age, gender, smoke, alcoholism, DM, CTD, CRF, CHD, blood urea nitrogen, temperature, respiratory rate, systolic pressure, diastolic pressure, heart rate, white blood cells, PLT, HGB, CURB-65, PSI, Vasoactive drugs, Accumulated dose of glucocorticoids.
